# COVID-19 Community Stabilization and Sustainability Framework: An Integration of the Maslow Hierarchy of Needs and Social Determinants of Health

**DOI:** 10.1017/dmp.2020.109

**Published:** 2020-04-21

**Authors:** Benjamin J. Ryan, Damon Coppola, Deon V. Canyon, Mark Brickhouse, Raymond Swienton

**Affiliations:** Baylor University, Texas; Shoreline Risk, LLC, Virginia; Daniel K. Inouye Asia-Pacific Center for Security Studies, Hawaii; Saint Louis University, Missouri; University of Texas Southwestern, Texas

**Keywords:** COVID-19, public health, recovery, risk management, societies

## Abstract

All levels of government are authorized to apply coronavirus disease 2019 (COVID-19) protection measures; however, they must consider how and when to ease lockdown restrictions to limit long-term societal harm and societal instability. Leaders that use a well-considered framework with an incremental approach will be able to gradually restart society while simultaneously maintaining the public health benefits achieved through lockdown measures. Economically vulnerable populations cannot endure long-term lockdown, and most countries lack the ability to maintain a full nationwide relief operation. Decision-makers need to understand this risk and how the Maslow hierarchy of needs and the social determinants of health can guide whole of society policies. Aligning decisions with societal needs will help ensure all segments of society are catered to and met while managing the crisis. This must inform the process of incremental easing of lockdowns to facilitate the resumption of community foundations, such as commerce, education, and employment in a manner that protects those most vulnerable to COVID-19. This study proposes a framework for identifying a path forward. It reflects on baseline requirements, regulations and recommendations, triggers, and implementation. Those desiring a successful recovery from the COVID-19 pandemic need to adopt an evidence-based framework now to ensure community stabilization and sustainability.

Nationwide restrictions on freedom of movement (“stay-at-home” orders, or “lockdowns”) have been imposed to contain the spread of coronavirus disease 2019 (COVID-19) and, by extension, avoid medical system capacity exceedance (colloquially termed “flattening the curve”).^1,2^ The COVID-19 virus’ rapid cycle of transmission, incubation, and presentation of symptoms means the 45-day period covered by the Presidential Guidelines will likely produce a measurable drop in the disease observed reproduction number (R0) in all but a few zones of exceptionally high incidence (“hotspots”).^3^ These measures, which carry an immediate and striking cost measured in millions of jobs lost, trillions of dollars (in social and economic support), a dramatic cessation of basic community function in all of the country’s incorporated cities, towns, and villages, are of questionable sustainability.^4^ The White House has empowered states, and by extension, local government officials, with decision-making authority to apply protection measures as they wish.^5^ It must be anticipated imminent decisions required to ease such restrictions will ultimately fall to these same state and local leaders. Without an effective framework to guide them, efforts to determine when it is “safe enough” to ease restrictions, what easing means, and whether or not any such actions are helping or harmful in the long term will be haphazard at best.

The novel SARS-CoV-2 virus driving the current pandemic, like 4 “common cold” coronavirus variants that have cycled through the global population for decades causing billions to suffer moderate respiratory tract illnesses, exhibits extremely high person-to-person transmissibility.^3,6^ With no viable vaccine anticipated for at least 12 to 18 months, and likely longer given the ambitiousness of that timeframe, it can be inferred that, at best, worldwide risk from COVID-19 will persist at positive levels for more than a year, and at worst for perpetuity if no vaccine is discovered.^7^ At the same time, politicization of the issue and an understandable sensitivity among those who fear the virus and are highly vulnerable have suppressed mainstream policy discussions about how and when lockdowns might be eased.^8^ This policy discussion suppression has occurred without accounting for the remarkable challenges associated with full eradication. Without broad consensus behind a viable national roadmap for recovery, states and communities will begin acting independently, and in the process, will re-establish the conditions by which geographic dispersion of the disease was able to occur at the outset of this crisis.

By applying an incremental approach to easing restrictions on movement, community leaders will be able to gradually restart the function of society within their jurisdiction while simultaneously maintaining most public health benefits achieved through current lockdown measures.^9^ Once stabilization of disease transmission has been achieved through the current lockdown, and effective multi-stakeholder coordination mechanisms are in place, incremental easing may be considered. From this starting point, ongoing infection control can be balanced with measured reopening of social networks and their associated economic drivers. Risk control remains the primary goal of all efforts, with an appreciation of the extent to which risk associated with COVID-19 infection differs between populations relative to other common hazards.

Crisis scholars have found that the behavior of our leaders, the mortality rate, the social trauma, and economic damage all do not play a large role in determining how long a crisis persists.^10^ Other scholars insist that the duration of a crisis is primarily a result of how well the accountability processes are managed.^11^ This study argues that the way the lockdown removal process is handled will have the most significant impact on how long the effects of the pandemic persist.

Resistance to this concept is likely among those who find no level of positive COVID-19 risk acceptable. However, sustained lockdown is unreasonable for multiple reasons, including the inability of socially and economically vulnerable populations to endure long-term livelihood interruption given the limits of Federal cash support; capacity limits of governmental relief capacities that are not designed to address simultaneous supply chain breaks in all states and territories; significant threat of psychological stress and injury caused by extended physical isolation; and other negative impacts on the ability of individuals to meet their basic human needs. Leaders will face overwhelming pressure to ease the imposed restrictions. With no existing strategy to guide such actions, the measures implemented will be reactionary, rushed, and lacking requisite analysis. We propose there is a safer incremental recovery process that is risk-conscious, accommodates variable population vulnerability, and quickly resumes access to basic human and societal needs.

## DISASTER RISK MANAGEMENT AND COVID-19

Disaster risk management operates with the assumption that society can never be risk-free. Actions, thus, are taken to prepare for disasters and mitigate their impact by reducing the likelihood of crisis and the magnitude of impacts on society. Preparedness ensures that a degree of thought and resources are dedicated to generating the knowledge and materials needed for response to and recovery from actualized disasters. Once a disaster occurs, the diverse disaster risk management community, including emergency managers, emergency services, public officials, businesses, nonprofit organizations, and others, act to address the immediate threats to life and property while beginning the process by which longer-term resumption of societal functioning may begin. In all societies, there exist more hazards than resources to mitigate them, resulting in the need to establish social acceptance of nonzero risk (as guided by the financial, social, physical, and other costs that individuals are unwilling to accept in exchange for any additional risk reduction benefits). Risk acceptability does change over time as understanding about individual risk increases and/or the different costs associated with disaster risk management options improve.

For COVID-19, those tasked with disaster risk management must base their risk assessments on epidemiological data that are incomplete, outdated, and in many cases inaccurate.^12^ Without a better understanding of infection rates across the greater population, and a focus on testing only those with specific symptoms, our understanding of the extent of the disease across the greater population (and likewise a more accurate basis of calculating the case fatality rate [CFR]) is limited.^13^ This in turn limits understanding of how the individual risk associated with COVID-19 compares to risks from hazards readily accepted by society.^14^ Without such information in hand, there is little option other than to apply a brutalist, overly conservative, and over-reactive approach to risk control to ensure that as many lives may be saved as possible.

From this philosophical outlook, nation-wide lockdown measures have emerged and generally appealed to the public. For many people, the risk basis for imposing such measures is statistically valid and may ultimately save their lives, while for others with lower risk factors such measures are and may eventually be viewed as excessive; however, no such confirmatory evidence-base for either condition yet exists. Given the lack of information and the high level of uncertainty, all actors perceive that the social costs are extremely high, and so the impacts of lockdowns are generally acceptable, regardless of their justification.

With each passing day, more is learned about how members of different demographic groupings are impacted by COVID-19. This is delineated on the basis of age, gender, medical history, behavior, access to healthcare, and many other disaggregation factors. There are no guarantees, but disaster risk management is based on probabilities and not certainties. We know not whether we will suffer a fatal accident each time we step into our automobiles, yet we assume the known risk because we have accepted it based on our understanding of its relative severity. As we increase our understanding of the relative risk of COVID-19 infection among different groups, individuals in those groups will begin to question their own necessity to remain in lockdown. Those tasked with managing the disaster will begin to question whether the cost of having a blunt lockdown approach, as it applies across all of society, is necessary or acceptable, given the risk reduction gained versus the extensive cost to the economy.

There are 2 parallel but unique goals of the restrictions imposed. The first is ensuring that infection rates remain low enough to maintain medical capacity (hospital beds, doctors, nurses, equipment, supplies) at adequate levels over time. The second goal is to maintain a sufficiently low rate of infection such that the most vulnerable populations are less likely to become exposed. Over two-thirds of those who become infected show no symptoms, and many more have only mild ones; however, for vulnerable populations, the disease represents a significant risk of mortality to themselves or to their elderly relatives.

Disaster risk managers experience a tension between their need to protect lives versus their need to ensure community viability. The definition of risk is “the effect of uncertainty on objectives,”^15^ and community objectives are diverse and include social and emotional health, education, prosperity, liberty, and many other things that are negatively impacted by ongoing COVID-19 mitigation measures. Management of risk, therefore, needs to address all community objectives, applying different requirements for different populations. A community does not, for instance, apply flood prevention requirements for all homes in a community because a part of that community lies in the floodplain. Once the extent of that floodplain, or any risk measure, is known, management options improve dramatically. It is vital to begin applying a risk management approach to the COVID-19 response and recovery.

## COVID-19 VULNERABLE POPULATIONS

The people at greatest risk from COVID-19 are older adults and people of any age with serious underlying medical conditions.^16^ According to the Centers for Disease Control and Prevention the older adults at risk of serious complications are those 65 years and older and the serious underlying medical conditions are: diabetes; liver disease; chronic lung disease or moderate to severe asthma; serious heart conditions; compromised immune system (eg, undergoing cancer treatment and poorly controlled AIDS or HIV); severe obesity (body mass index [BMI] of 40 or higher); and undergoing dialysis (chronic kidney disease).^17^ An important consideration is that approximately 45% (range, 37% to 52%) of hospitalizations are people aged <55, and this combined with the health resources required to cater for the elderly is adding further capacity strain to the health system.^18^

People with underlying health conditions need to take particular care in protecting themselves from COVID-19. For example, in the United States, a Centers for Disease Control and Prevention report found 94% of patients who died had at least 1 underlying condition.^19^ Protection can be achieved through washing hands; cleaning and disinfecting high-touch surfaces; and social distancing, including staying at home, avoiding crowds, gatherings, travel, and contact with persons who are ill.^19^ This should be complemented by having a 2-week supply of food and necessities and 30 days of medication.^20^ Understanding these data and the most vulnerable groups provides an evidence-based and -driven opportunity for decision-makers to consider whole-of-society needs and requirements for dealing with the COVID-19 crisis with a focus on mitigating long-term societal impacts.

## UNDERSTANDING AND INCORPORATING HUMAN AND SOCIETAL NEEDS

Identifying and understanding the system of human needs is critical for defining future COVID-19 response and recovery strategies. Subjective needs will determine both individual behavior and effective response levels.^21^ Maslow’s hierarchy of needs (Table 1) provides a framework for understanding these needs, system impacts on society, and what motivates humans.^22^ The basic physiological needs are the foundation of the hierarchy, which include having water, food, and shelter met at a certain degree. In Maslow’s theory, the more the physiological needs are satisfied, the more the person will attempt to satisfy the safety and security needs, and so on.^23^ However, the COVID-19 lockdowns are compromising safety needs, such as access to employment and resources, which can comprise the desire for people to achieve the next level of needs related to social, esteem, and self-actualization.

**TABLE 1:** Maslow Hierarchy of Needs and Impact of COVID-19 Lockdown

Goal (Basic Need)	Examples of Requirements	Possible COVID-19 Lockdown Impact on Individuals and Society
1. Physiological needs	Breathing, homeostasis, water, sleep, food, sex, clothing, shelter, mobility	Less mobility, food access issues, and for some people shelter may be affected.
2. Safety needs	Employment, resources, property, health, stability, and security	Increased unemployment, reduced access to resources, and individual stability impacted due to uncertain future. Security issues may increase at household/domestic level.
3. Social needs	Love, affection, family, friends, relationships, and belongingness	Access to family and friends impacted.
4. Esteem needs	Recognition, respect, achievement, self-confident, and self-worth	Self-worth questioned as people become unemployed and have an uncertain future.
5. Self-actualization	Creativity, acceptance of facts, morality, and problem solving	Little to no impact.

Adapted from Ryan (2018).^24^

Decision-makers must urgently integrate the Maslow hierarchy of needs into current policy to resolve whole-of-society COVID-19-related crises, or else cause unnecessary long-term societal harm and societal instability. For example, Somali piracy in the Gulf of Aden was driven by the inability of Somalis to meet their physiological and psychological needs on land.^25^ As the COVID-19 lockdowns continue, the likelihood of this occurring will significantly increase, particularly for the younger and healthy working population, who are at low risk from the virus. This tipping point will be when they see their current needs as unmet and their opportunity for future growth and potential disappear.^26^

To address this challenge a “Society Needs” approach must be taken, and the first step is the application of disaster management principles and practices. Decision-makers must understand that actions of individuals in an emergency are typically consistent with the hierarchy of goals in Maslow’s motivation theory.^21^ For example, people make decisions based on perceptions of their physiological needs before considering their safety needs. People will shelter if they have resources for their basic needs while the threat continues. However, when this threshold is compromised, there may be societal consequences as people may take it upon themselves to meet their needs. Although Maslow’s hierarchy of needs is not a clear linear progression, it offers a multilayered model where COVID-19 management activities could meet the needs of the entire society, particularly if integrated with the social determinants of health.

The social determinants of health, are internationally recognized as key to any healthy society and must be integrated with the Maslow’s hierarchy of needs when considering responses to the pandemic. These factors include the conditions in which people live and work and the broader forces that influence daily life, such as economic policies, development agendas, social norms, social policies, and political systems.^27^ More specifically, these factors include health-care services, water and sanitation, lifestyle, education, and working and living conditions.^28^ Aligning decisions with these factors will ensure the approach is based on “Society Needs.” This is vital to making certain needs of all segments of society are catered to and met while managing the crisis. This must inform the process of incremental easing of lockdowns to facilitate the resumption of community and social foundations such as commerce, education and employment in a manner that protects those most vulnerable to COVID-19.

## COMMUNITY STABILIZATION AND SUSTAINABILITY FRAMEWORK

Communities are diverse in every regard, and there is no one-size-fits-all approach to easing of COVID-19-based restrictions that may be applied. Using a framework model, however, leaders and other disaster risk management stakeholders can identify for their own community a path forward that is acceptable to constituents with regard to both the acceptability of risk over time, and the reduction in crisis control costs. This could be guided, for example, by a COVID-19 color-coded risk management framework^29^ using 3 parts:

### 1. Baseline Requirements

Incremental easing will be possible only when a minimum set of requirements has been satisfied. National level requirements will include establishment of an open-data disaggregated public health tracking database, technical and financial support for testing and tracing, and social programming to ensure vulnerable populations are able to maintain longer-term protection measures (extended shelter-in-place). Systems will also need to be in-place to protect the most vulnerable populations. At the local and state level, this will include establishment of crisis management decision-making bodies, passage of emergency laws and ordinances, enforcement mechanisms to support changing control measures, and enhanced treatment, care, and services for vulnerable populations.

### 2. Regulations and Recommendations

Regulations and recommended definitions must be developed that are applicable to a series of increasing Health Condition (HEALTHCON) designations. Social distancing requirements would ease incrementally, guided by sector- and activity-specific designations: for instance, the number of patrons per square feet of restaurant or store space; the wearing of personal protective items, such as masks; the maintenance of 6 feet social distancing in public places; limits on the number of people who can congregate in parks or indoor spaces; and requirements to sanitize surfaces or equipment at designated intervals. Recommendations would apply to groups based on their vulnerability. For some groups, vulnerability is so great as to negate any easing of social distancing recommendations. For others, recommendations will enable increased movement and activity in the community, but with personal protective equipment or avoidance of higher-risk activities. Unlike requirements, which must be enforceable, recommendations are voluntary.

### 3. Triggers

Quantitative data-based triggers must be established to determine when a community is able to shift between different HEALTHCON levels. Some will apply only to specific facilities or groups (eg, if a student in a class tests positive for COVID-19), and some will apply to the entire community (eg, if a threshold level of infections is identified in the community). Increased easing would come only with sustained control at designated levels of community exposure (ie, established numbers of people infected and/or exposed), which are affiliated with accessible medical capacity. It is important to note that HEALTHCON for a community will be influenced by neighboring communities, and by conditions noted state-wide.

A trigger/threshold for reopening could be when cases peak and there are sustained declines in new cases, and hospitals are able to safely treat all patients requiring hospitalization.^30^ When achieved, schools, for example, could incrementally reopen by: staggering the beginning and end of the school day; not having assemblies, sports games, and other events that create crowded conditions; space is provided for children’s desks to be at least 1 meter apart; and having a teaching model that creates space and avoids unnecessary touching/contact.^31^ This would need to be closely monitored, and if after 14 days, the number of cases continues to decline, steps could be made to further ease restrictions by having staggered assemblies and starting school at the same time for all children. If this continues to be effective for the next 14 days, the school could then resume activities. Another option that could be applied is the Taiwan “7-2-7” model.^32^ If 2 students in 1 class are infected within 7 days, the class is suspended for 7 days.^32^ Throughout this process there would need to be constant monitoring, and if the cases increase and/or hospital capacity reduces, the protective measures could be incrementally introduced.

Workplace re-openings may need to be more aggressive for hourly and blue-collar workers. This could potentially be facilitated by staff wearing extra personal protect equipment, such as face coverings. The need for this more aggressive approach is that white collar jobs are more manageably accomplished remotely, which also allows those employees to retain salaries and benefits. However, hourly and blue-collar workers are more vulnerable as they usually lack access to telework options. This increases their need to return to work, and if not supported in the safest possible manner, could put them and society at greater risk. This highlights the need for data-based triggers to determine when certain sections of a community are able to shift between different HEALTHCON levels.

While artificial intelligence solutions are ideally suited to tracking data triggers, most agencies do not have access to this technology and a simpler approach is necessary. A systematic tool that could be used to drive and steer a collaborative government approach would be application of the United Nations Office for Disaster Risk Reduction’s public health system resilience scorecard.^9^ There are sections of the scorecard, directly relevant to pandemic preparedness, response, and recovery, that can be rapidly applied and would generate the conversations required to identify triggers and strategies and thresholds for easing lockdowns while ensuring the safety of vulnerable populations. This should be completed by interdisciplinary discussions about the framework requirements outlined in this study along with a pandemic tabletop exercise to test the scenarios and assumptions with the needs of the whole society at the forefront of consideration.^33^ Applying this approach would ensure a collective decision for determining when it is “safe enough” to begin easing lockdowns.

## IMPLEMENTATION CONSIDERATIONS

A collaborative governance approach is required to develop and implement incremental easing of COVID-19 lockdowns due to the wide-range of disciplines, organizations, and sectors involved, and the need for a collective decision on when it is “safe enough”.^9,34^ Perspectives must be sought from beyond disaster management stakeholders to allow key community and private organizations, such as universities, primary health and transport companies, to help solve this ethical and societal challenge.^35^ The process could be rapid and include, for example, convening an urgent local emergency planning committee meeting to examine the data, people most at risk, and identify thresholds for determining when “safe enough.” The groups would engage in comprehensive and shared planning, allow communication across multiple levels, and facilitate pooling of resources to implement the strategies for easing COVID-19 lockdowns.^36^

As leaders formulate strategies that transition them out of this crisis, they do well to heed the principles of intersectoral collaboration and risk management.^36–38^ This includes considering the primary hazard and all its ripple effects, engaging with all agencies and sectors, and expanding beyond the traditional health sector focus of this pandemic to consider the community-wide implications of COVID-19 response strategies. A key step for considering the social and economic dimensions of this crisis is focusing on the most vulnerable. This can only be achieved by designing policies that support the provision of health, unemployment insurance, and social protections while bolstering business to prevent bankruptcy and job loss.^39^ This could be guided, for example, by developing a COVID-19 color-coded risk management framework informed by the public health system resilience scorecard, Maslow hierarchy of needs, and the social determinants of health.^29^ Achieving this would demonstrate a mature whole-of-society-needs approach to COVID-19 that would go a long way toward mitigating post-crisis accountability processes.

## CONCLUSION

As we navigate a path to stabilization of and recovery from the ongoing COVID-19 pandemic, political leaders must explore ways to minimize social, community, and economic repercussions of protective measures by exploring effective yet socially acceptable strategies for easing restrictions. Major crises of any duration, scope, and intensity challenge leadership capacity, and the COVID-19 pandemic is perhaps the greatest crisis of our time. Decision-makers face the difficult task of transitioning the nation, states, municipalities, and organizations out of protective measures and into longer-term recovery. For this crisis, such efforts are most challenging given the potential recriminations, accusations, and fallout that can result from unexpected outcomes. Leaders that use a well-considered framework with an incremental approach to easing restrictions will be able to gradually restart the function of society within their jurisdiction while simultaneously maintaining most public health benefits achieved through current lockdown measures. A COVID-19 color-coded risk management framework informed by the public health system resilience scorecard, Maslow hierarchy of needs, and the social determinants of health would provide the systematic mechanism required for this to occur. Achieving this would demonstrate a mature whole-of-society-needs approach to COVID-19 that would go a long way toward mitigating post-crisis accountability processes by answering the key question, “When is it safe enough to begin community stabilization and incrementally ease lockdown provisions, and how will we know if our efforts are working?”.
